# Physiotherapeutic Interventions in Diabetic Foot Ulcer Management: A Case Report

**DOI:** 10.7759/cureus.55244

**Published:** 2024-02-29

**Authors:** Vaishnavi R Waghe, Vrushali Athawale

**Affiliations:** 1 Physical Medicine and Rehabilitation, Ravi Nair Physiotherapy College, Datta Meghe Institute of Higher Education and Research, Wardha, IND; 2 Oncology Physiotherapy, Ravi Nair Physiotherapy College, Datta Meghe Institute of Higher Education and Research, Wardha, IND

**Keywords:** diabetes mellitus, rehabilitation, wound debridement, physiotherapy, diabetic foot ulcer

## Abstract

Diabetic foot ulcers (DFUs) represent prominent complications arising from diabetes mellitus, characterized by the development of severe and persistent wounds involving the loss of epidermal and/or dermal layers, with the potential to extend into subcutaneous and underlying tissue structures. In the presented case, a 62-year-old male patient presented with complaints of pain and the emergence of blisters on the right foot, marked by an insidious onset and gradual progression in size, ultimately leading to ulcer formation subsequent to blister rupture. The patient had a medical history spanning 25 years of diabetes mellitus, accompanied by diminished range of motion and muscle strength in the affected foot. The primary goals in the management of DFU encompass addressing muscular weakness, skin manifestations, and any associated underlying health comorbidities. Central to this management approach lies the incorporation of physical therapy and rehabilitation modalities. The rehabilitation regimen designed for the patient was tailored to include targeted resistance exercises, infrared radiation therapy, and sensory integration therapy. Outcome measures, including the Numeric Pain Rating Scale (NPRS), World Health Organization Quality of Life questionnaire (WHO-QOL), and Diabetic Foot Self-Care Questionnaire (DFSQ-UMA), demonstrated substantial improvements, reflecting enhanced activities of daily living. This case underscores the pivotal role of incorporating physiotherapy into a comprehensive multidisciplinary approach for optimizing the management of DFUs. Such integration aims to improve patient outcomes and overall quality of life.

## Introduction

Diabetes mellitus represents a medical condition characterized by a widespread occurrence, significant morbidity, and notable mortality rates, to the extent that it is acknowledged as a global epidemic [1]. A study conducted by Shailesh K. Shahi reported that the prevalence of diabetic foot ulcers (DFUs) among diabetic patients was 14.30% (95% CI=11.67-16.94) [2]. DFUs stand out as primary complications arising from diabetes mellitus, presenting as severe, chronic wounds characterized by the loss of epidermal and/or dermal layers, with potential penetration into subcutaneous and underlying tissue structures [3,4]. The underlying etiology of DFUs is categorized into three types: purely neuropathic (35%), purely ischemic (15%), and mixed neuroischemic (50%) [5]. The pathophysiology of the DFU is attributed to a triad consisting of neuropathy, trauma with subsequent infection, and arterial occlusive disease [6]. These classifications hinge upon the presence or absence of peripheral neuropathy (PN) and associated sensory loss (neuropathic), peripheral artery disease (PAD) (ischemic), or both (neuroischemic) [7]. Various risk factors contribute to the development of DFUs, including age, sex, race, socioeconomic status, obesity, smoking, cardiovascular diseases, chronic kidney disease, retinopathy, and other associated comorbidities [8].

DFUs are further complicated by infections, peripheral vascular diseases, or diabetic neuropathies. These complications may lead to reduced joint mobility, alterations in gait, and increased pain, all of which diminish the quality of life and impede the performance of daily activities [9,10]. A study conducted by Medeiros S concluded that despite the high prevalence and risks associated with DFUs, there is an increasing acknowledgment of the pivotal role physiotherapy can assume in their management. Physiotherapeutic interventions, encompassing exercise, electrotherapy, and manual therapy, hold considerable potential in augmenting the healing process and ameliorating the complications linked with DFUs [11]. Physical activity exerts a noteworthy impact on diminishing glycated hemoglobin levels, with the amalgamation of aerobic and resistance exercises demonstrating heightened efficacy in contrast to engaging in either aerobic or resistance exercise in isolation. Additionally, exercise contributes to the enhancement of the ankle-brachial index [12].

According to a study conducted by Aydın, engaging in appropriate aerobic, strengthening, balance, and flexibility exercises can mitigate the risk of diabetic wound development. Moreover, wound healing may be enhanced through exercise regimens and physical therapy interventions. Furthermore, it is crucial to mobilize patients with DFUs to mitigate complications associated with prolonged immobilization. Based on the findings of the literature review, rehabilitation emerges as a safe and effective strategy for both preventing and managing DFUs [13]. This case report scrutinizes the effects of a customized physiotherapy regimen in addressing a DFU in a patient with longstanding diabetes, offering valuable insights into the prospective advantages of these interventions in enhancing patient outcomes.

## Case presentation

The 62-year-old patient was right one month ago when he manifested blisters on the right foot, characterized by an insidious onset and gradual progression in size, culminating in ulcer formation subsequent to the bursting of these blisters. The associated pain, described as insidious and gradually progressive pricking-type discomfort, was accompanied by difficulty in ambulation. Additionally, the patient reported intermittent low-grade fever, alleviated with medication. Despite seeking prior medical attention and attempting home remedies, the symptoms persisted, prompting the patient to seek further management at Acharya Vinoba Bhave Rural Hospital. The patient has an established medical history of diabetes mellitus spanning a duration of 25 years, alongside a concurrent diagnosis of hypertension. Further investigations included ultrasonography of the right lower limb doppler, revealing a dilated great saphenous vein with involvement of the small saphenous vein. For which the patient underwent an intervention involving wound debridement for the right DFU. On the Numerical Pain Rating Scale, the patient reported a pain score of 8/10 during movement and 2/10 at rest at the debridement site. Pain exhibited exacerbation with movement and was mitigated by rest and medication, without diurnal variations in symptoms noted.

Clinical findings

Upon obtaining informed consent, a comprehensive patient assessment was conducted. Consistent with the provided medical history, the patient disclosed a two-decade history of diabetes mellitus concomitant with hypertension. Subsequently, a wound debridement procedure was executed for the right foot diabetic ulcer. Following the intervention, the patient reported heightened pain in the right foot, notably exacerbated during weight-bearing activities such as walking with an altered gait pattern. The examination, conducted with the patient in a supine position, revealed diminished muscle strength in the right lower limb. A local examination identified Grade II tenderness at the site of the ulcer and showed a decreased range of motion in the right ankle joint as presented in Table 1. The patient had decreased strength in the right foot as shown in Table 2.

**Table 1:** ROM of right ankle joint before and after the treatment ROM: Range of motion; AROM: active range of motion; PROM: passive range of motion

ROM	Measurement	Before treatment		After treatment	
		AROM	PROM	AROM	PROM
Ankle joint	Dorsiflexion	0˚-5˚	0˚-10˚	0˚-20˚	0˚-25˚
	Plantarflexion	0˚-5˚	0˚-10˚	0˚-15˚	0˚-20˚

**Table 2:** MMT of right ankle muscles MMT: Manual muscle testing; 2/5: Full ROM gravity eliminated; 4/5: Full ROM against gravity, moderate resistance; ROM: range of motion

MMT	Measurement	Before treatment	After treatment
Ankle	Dorsiflexors	2/5	4/5
	Plantarflexors	2/5	4/5
	Invertors	2/5	4/5
	Evertors	2/5	4/5

Therapeutic intervention

**Figure 1:**
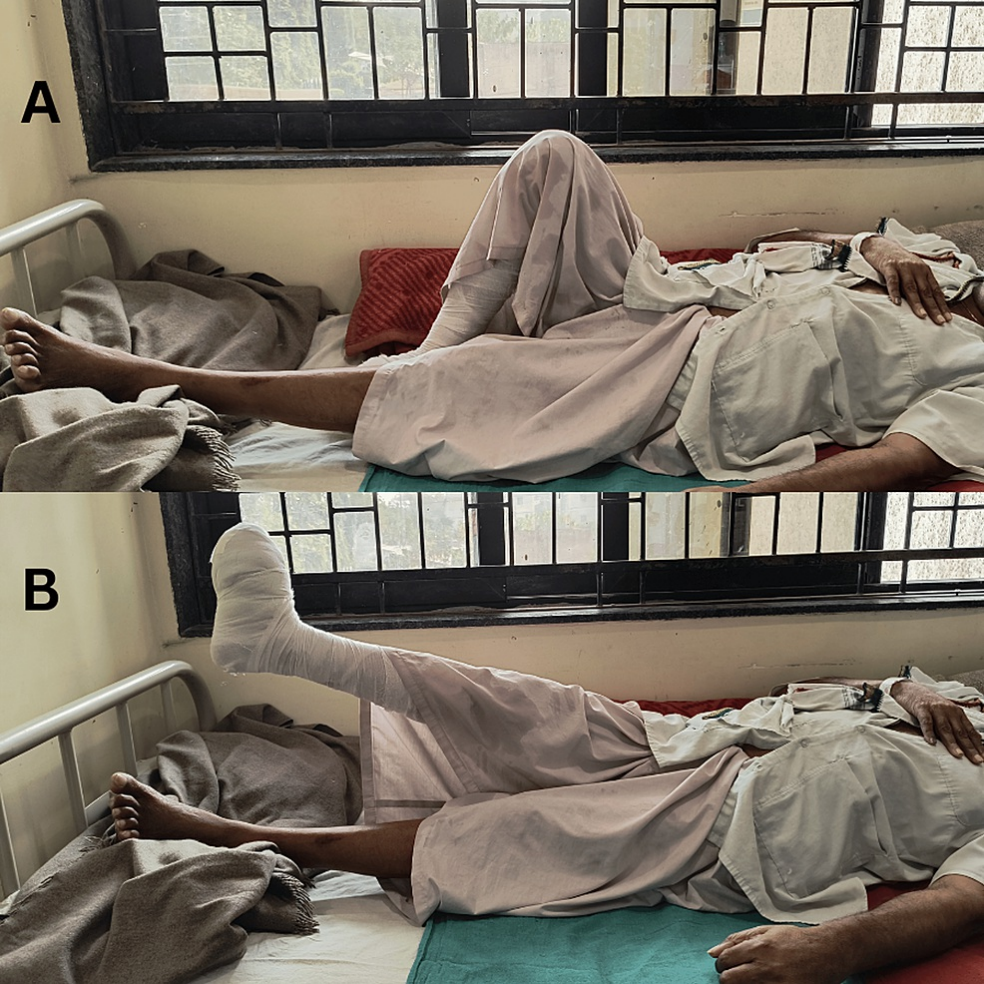
Patient performing exercises: (A) heel slides, (B) straight leg raise

Table 3 shows the therapeutic intervention given for four weeks. Patients performing heel slides and straight leg raising are shown in Figures 1A, 1B.

**Table 3:** Therapeutic intervention given for four weeks

Goals	Intervention (1-4 weeks)	Repetition
To educate about the precautions after wound debridement, complications, wound care and importance of physiotherapy	Patient education	-
To prevent hospital acquired diseases, to maintain functional capacity and to prevent deep vein thrombosis	Breathing exercises: Pursed lip breathing, Thoracic expansion exercise with upper limb mobility with 5 sec hold, ankle pumps	10 repetitions, 1 set
To make patient independent on bed	Bed mobility exercises: supine to side lying- side lying to sitting	-
Wound care	Infrared radiation	10 minutes
Improving sensory awareness and promoting protective sensation	Sensory integration therapy	10 minutes
To increase strength of unaffected extremities	Strengthening of unaffected lower limb and bilateral upper limb: heel slides with 1 kg weight cuff, dynamic quads, biceps curls with 1 kg weight cuff, upper limb mobility with flexion-extension, abduction-adduction with 1 kg weight cuff	5 repetitions in first week gradually increasing to 8- 12 repetitions
To make patient ambulate	Ambulation with walker on non-weight bearing of affected leg, pivot transfer while walking	2 rounds
To increase strength of right affected extremity	Strengthening of affected lower limb: ankle toe movements, static quadriceps, static hamstrings, dynamic quads, initiation on partial weight bearing	5 repetitions in second week gradually increasing to 8- 12 repetitions

Follow-up and outcomes

Table 4 shows the outcome measures pre-rehabilitation and post-rehabilitation after four weeks of rehabilitation.

**Table 4:** Outcome measures pre and post rehabilitation WHO-QOL: World Health Organization Quality-of-Life Scale

Outcome Measures	Pre-rehabilitation	Post-rehabilitation
Numerical Pain Rating Scale	7/10	3/10
WHO-QOL	48/100	72/100
Diabetic foot self-care questionnaire (DFSQ-UMA)	38/100	79/100

## Discussion

This case report delves into the effectiveness and implications of physiotherapeutic interventions in managing DFUs, a consequential complication of diabetes mellitus known for inducing chronic wounds that present formidable challenges in treatment and wound healing. Given the multifaceted nature of DFUs, a holistic approach becomes imperative, encompassing not only wound care but also the underlying pathophysiological mechanisms governing ulcer initiation and progression. In this study, physiotherapy played a significant role in wound care by administering infrared radiation. Moreover, to enhance musculoskeletal function and mobility, the patient received resistance exercises and stretching exercises. Additionally, sensory integration therapy was provided to address neuropathic impairment in the patient. The integration of physiotherapy interventions in this context likely contributed to the observed improvements in range of motion, muscle strength, and sensory awareness, thereby promoting enhanced functional abilities and mobility for the patient.

A study conducted by Boyd highlighted that diabetes mellitus leads to the onset of peripheral neuropathy, which manifests in diverse forms, including sensory, focal/multifocal, and autonomic neuropathies. It is noteworthy that more than 80% of amputations occur following foot ulceration or injury, frequently attributable to diabetic neuropathy [14]. Peripheral neuropathy may engender muscle weakness and diminished reflexes, particularly at the ankle, precipitating alterations in gait. Concurrently, foot deformities, such as hammertoes and midfoot collapse, may ensue. The insensitivity to pressure or injury in numb areas of the foot may give rise to unnoticed blisters and sores. Left untreated, these may escalate into bacterial and fungal infections, along with foot ulcers, ultimately culminating in the necessity for amputation [15]. The primary objectives of diabetic foot care involve a comprehensive integration of preventive strategies. This includes patient education, active participation, adherence to physiotherapy interventions, and maintaining strict glycemic control. Regular monitoring of skin, foot, and nail health is crucial for preventing complications and enhancing overall diabetic foot wellness. As demonstrated in our study, adherence to exercise regimen facilitated faster recovery [16].

A comprehensive meta-analysis conducted by Cochrane, encompassing 14 randomized controlled trials (RCTs) and published in 2009, revealed that exercise interventions along with medications yielded a reduction in HbA1c levels by 0.6% (95% CI = 0.3% to 0.9%) when compared to non-exercise counterparts [17]. Integrated physical exercise is presumed to activate a broader spectrum of skeletal muscle mass in contrast to individual modalities such as aerobic or resistance exercise. Given adequate duration and intensity, such comprehensive exercise regimens are anticipated to instigate adaptive responses across a larger expanse of muscle mass, including the associated microvasculature [18]. Olver and Laughlin have proposed that optimizing skeletal muscle fiber recruitment throughout the entire body in exercise programs holds potential for enhancing glycemic control [19].

## Conclusions

The case report presented underscores the critical role of physiotherapeutic interventions coupled with adherence to an exercise regimen in the comprehensive management of DFUs. Through a personalized regimen incorporating various modalities including wound care, exercise prescription, and patient education, significant enhancements in wound healing, pain management, and functional outcomes were noted. This highlights the importance of integrating physiotherapy within the multidisciplinary approach to optimize the management of DFUs, thereby promoting improved patient outcomes and quality of life.
